# Music-Based Therapy for the Treatment of Perioperative Anxiety and Pain—A Randomized, Prospective Clinical Trial

**DOI:** 10.3390/jcm13206139

**Published:** 2024-10-15

**Authors:** Shiv K. Goel, Valdemir Kim, Jeremy Kearns, Daniel Sabo, Lynsie Zoeller, Coleen Conboy, Nicole Kelm, Ann E. Jackovich, Jacques E. Chelly

**Affiliations:** 1 Department of Anesthesiology and Perioperative Medicine, University of Pittsburgh, Pittsburgh, PA 15261, USA; goelsk@upmc.edu (S.K.G.); kimv@upmc.edu (V.K.); kearnsjt@upmc.edu (J.K.); sabodp@upmc.edu (D.S.); zoellerl@upmc.edu (L.Z.); 2 UPMC Shadyside Hospital, Pittsburgh, PA 15232, USA; conboycm@upmc.edu (C.C.); bachmannk@upmc.edu (N.K.); 3 UPMC McKeesport Hospital, McKeesport, PA 15132, USA; aemjackovich1@comcast.net

**Keywords:** music-based intervention, anxiety, depression, catastrophizing, ambulatory surgery

## Abstract

**Background**: Music-based intervention has been advocated as a nonpharmacologic approach for the perioperative control of pain and anxiety in surgical patients. However, its impact on patients with preoperative anxiety has not been clearly established. Our study aimed to examine the impact of music-based intervention administered before, during, and after surgery on postoperative opioid consumption and pain levels, as well as preoperative anxiety, depression, and pain catastrophizing. We hypothesized that, compared to a control group, music-based intervention would be effective in reducing opioid requirements and mood disorders. **Methods**: This study was a single-center, prospective, single-blinded, randomized controlled trial. Inclusion criteria isame-day or observation surgery. Exclusion criteria included American Society of Anesthesiologists physical status IV, use of spinal anesthesia, PROMIS Anxiety T-scores ≤ 57.4 and ≥74.1, preoperative chronic opioid use, transgender surgery, and history of drug or alcohol abuse. Music-based intervention was developed by a certified music therapist. Each patient was randomized to receive standard of care (SC) or SC plus music-based intervention before, during, and after surgery. The primary end point was postoperative oral morphine equivalents (OMEs) over 5 days following surgery using the area under the curve (AUC)Secondary end points were PROMIS Anxiety, PROMIS Depression scores Pain Catastrophizing Scale scores, postoperative nausea and vomiting, time of hospital discharge, and patient satisfaction (0 = totally unsatisfied to 10 = completely satisfied). **Results**: A total of 75 patients were randomized to a music-based intervention (*n* = 33) or control (*n* = 42) group. Patients in the music-based intervention group consumed 56.7% less opioids than those in the control group (AUC was 2.8 in the music-based intervention group vs. 6.4 in the control group, absolute standardized mean difference (aSMD) = 0.34 (−0.17, 0.85)). No difference in pain scores was recorded between groups. Music-based intervention also reduced anxiety on postoperative day (POD)2 (aSMD = 0.38 (−0.16, 0.91)), depression on POD2 (aSMD = 0.31 (−0.23, 0.84)) and POD4 (aSMD = 0.24 (−0.29, 0.77)), and pain catastrophizing on POD1 (aSMD = 0.24 (−0.3, 0.77)). **Conclusions**: Our data support the use of music-based intervention to reduce postoperative opioid requirements. Music-based intervention may also reduce anxiety, depression, and pain catastrophizing.

## 1. Introduction

Up to 50% of patients report experiencing a certain degree of anxiety, depression, or catastrophizing before surgery [1], and up to 80% report pain after surgery [2]. Furthermore, the presence of mood disorders, especially anxiety, has been established as a factor leading to increased postoperative pain and opioid consumption [3,4,5,6,7,8].

In surgical patients, inappropriate management of postoperative pain has been associated with several unfavorable outcomes, including increased morbidity, the development of chronic pain, delayed surgical recovery, and persistence of opioid consumption and opioid use disorder [9]. The current recommended approach to perioperative pain management is multimodal analgesia, which includes non-steroidal anti-inflammatory drugs, acetaminophen, regional nerve blocks, and opioids [10]. A study of 36,177 adults in the US in 2017 reported an approximate 6% incidence of new persistent opioid use after minor and major surgeries. The same study identified anxiety as a significant risk factor [11]. Therefore, considerations have been given to the use of nonpharmacological complementary approaches as a way to control preoperative mood disorders and therefore improve pain control [12,13,14,15,16,17,18,19].

Among nonpharmacologic approaches to the perioperative management of pain and mood disorders, music-based intervention is a method that could be particularly valuable in health care, as it is inexpensive, has no known side effects, and does not require patients to have previous formal experience with music. Music-based interventions are used in health care to help reach various health-promoting goals and are provided by certified music therapists, health care professionals, musicians, and others. Music-based interventions where patients listen to pre-recorded music are a passive method often used by music therapists to relax a patient, regulate activity and tension, and positively influence the patient’s mood and motivation. This is why a licensed music therapist is needed to effectively establish a therapeutic relationship between the music and the patient, while also preventing side effects like anxiety. The current literature suggests that music-based intervention is an effective approach to controlling pain and anxiety [16,20,21,22,23,24,25,26]. Although a meta-analysis reported positive effects of music-based intervention, the high degree of heterogeneity in study designs has led to inconsistent conclusions.

Our study was designed to examine the impact of music-based intervention administered before, during, and after surgery on postoperative opioid consumption and pain levels, as well as preoperative anxiety, depression, and pain catastrophizing. This study also evaluated patient satisfaction and recovery outcomes. We hypothesized that, compared to a control group (standard of care), the use of music-based intervention would be effective in reducing opioid consumption in patients with preoperative anxiety and that a correlation exists between opioid consumption and anxiety.

## 2. Materials and Methods

### 2.1. Study Design and Participants

This study was a single-center, prospective, single-blinded, randomized controlled trial. Before subject recruitment was initiated, the study protocol was reviewed and approved by the University of Pittsburgh Institutional Review Board (STUDY21110130) and registered at clinicaltrials.gov (NCT05263635).

The inclusion criteria were patients having same-day surgery including breast surgeries, open inguinal hernia repairs, and laparoscopic or robotic surgeries. The patients received a single nerve block and underwent either general anesthesia or monitored anesthesia care. The patients’ ages ranged from 18 to 80 years old; their American Society of Anesthesiologists (ASA) physical statuses were I, II, or III; and they presented moderate anxiety (PROMIS Anxiety Short Form 8 questionnaire T scores ≥ 57.4 and ≤74.1).

The exclusion criteria were ASA physical status IV, use of spinal anesthesia, significant anxiety with PROMIS Anxiety Short Form 8 T-score of <57.4 or higher than 74.1, preoperative chronic use of opioids, and history of drug or alcohol abuse.

### 2.2. Enrollment Procedures

In the same-day surgery unit, a trained research coordinator approached patients who met the eligibility criteria at least two hours before surgery. Patients interested in participating were asked to sign a HIPAA-compliant informed consent document. After providing written informed consent, each patient was asked to complete the PROMIS Anxiety Short Form 8 questionnaire. Patients with mild to moderate anxiety (T-scores ≥ 57.4 and ≤74.1) [27] were randomized to either a group who would receive music-based intervention or a control group (no music-based intervention) using a computer-generated randomization scheduled. Baseline data before randomization also included medical history, pain rated using a verbal scale (0 = no pain to 10 = the worst possible pain), and pain medication use (including opioids). In addition, each patient was also asked to complete PROMIS Emotional Distress–Depression Short Form 8 (PROMIS Depression) and a Pain Catastrophizing Scale questionnaire [28].

Computer-generated, blocked randomization was performed with a 1:1 allocation ratio. Information about the allocation was stored in opaque envelopes.

### 2.3. Music-Based Intervention

The music-based intervention was developed by a certified music therapist (license registered by the medical board of Pennsylvania) and was based on flowing rhythm approximately 60–80 beats/minute, and was played by string instruments [29,30]. Each patient was offered to choose between classical, jazz, piano, guitar, or lo-fi music (music with a laid-back and chilled-out vibe). Lo-fi music often features slow tempos, simple melodies, and a repetitive nature that allows the listener to relax and unwind. Lo-fi music also heavily relies on the use of samples, particularly from vinyl records, to create its signature sound (examples can be found at soundscapehq.com). The music included in each category is listed in Appendix A. An MP3 player connected to headphones played the music-based intervention at a volume of approximately 45 decibels during three sessions: in the preoperative area, immediately following induction of anesthesia in the operating room, and in the post-anesthesia care unit (when subjects were awake and responsive).

### 2.4. Anesthesia

Surgery was performed under sedation (breast surgery, inguinal hernia repair) or general anesthesia. Prior to surgery, peripheral nerve blocks were performed, either a paravertebral nerve block in patients undergoing breast surgery [31,32] or quadratus lumborum approach (QL 2) in patients undergoing abdominal surgery [33]. The nerve blocks were performed prior to surgery in the same surgery units by a member of the acute interventional pain service. Furthermore, no opioids were administered prior to anesthesia and surgery.

### 2.5. Follow-Up

In the recovery room, postoperative pain level, analgesics, nausea, and vomiting were recorded until discharge. Prior to discharge, each patient was also asked to complete satisfaction questionnaires. After discharge, each patient was asked to complete questionnaires and report mood symptoms and medication use daily for the first five postoperative days (PODs). Specifically, they were instructed to fill out the PROMIS Anxiety, PROMIS Depression, and PCS surveys and record their levels of pain, anxiety, and pain medication use over the previous 24 h. Research electronic data capture (REDCap) was used to collect all the information.

### 2.6. Outcome Measurements

The primary end point was opioid consumption. Secondary end points included PROMIS Anxiety, PROMIS Depression, PROMIS sleep interference, and pain catastrophizing scores; pain and opioid consumption on PODs 1, 2, 3, 4, and 5; total opioid consumption over the first 30 days; incidence of postoperative nausea and vomiting (PONV); time of hospital discharge; and patient satisfaction scores (0 = totally unsatisfied to 10 = completely satisfied).

### 2.7. Statistical Analysis

Descriptive statistics were calculated using means and standard deviations for continuous data and counts and percentages for categorical data. Non-normal continuous data were described using medians and interquartile ranges. Differences between treatment groups prior to starting the treatment were tested on continuous data using T-tests and Mann–Whitney U tests, and categorical differences using Chi-squared tests and Fisher’s exact tests. Missing values were removed from all denominators and statistical testing. *p* values < 0.05 were considered significant.

Differences between means of the treatment vs. the control group were assessed using the absolute standardized mean difference (aSMD). An aSMD ≥ 0.2 indicates that the standardized means of these two groups are different [33,34]. R software (version 4.3.1, R Core Team, 2023) was used for data management and analysis. The *TOSTER* package was used to calculate SMDs, *DescTools* was used to calculate AUCs, and *halfmoon* was used to construct love plots.

The primary outcome was postoperative (POD) oral morphine equivalents (OMEs) over 5 days following surgery by calculating area under the curves (AUCs) using the trapezoid method. Power analysis indicated that 30 patients per group were required to establish a difference of 30% between the groups. Percent differences of medians and aSMDs were calculated to compare between-group differences. Love plots over time were created to visualize aSMDs over PODs. Secondary outcomes included pain over 5 days following surgery by calculating area under the curves (AUCs) using the trapezoid method, postoperative opioid consumption on POD 1–5, patient’s satisfaction surveys, postoperative length of stay, and level of nausea were compared using aSMDs and percentage differences of medians. Spearman correlations were calculated between total OME in the postoperative period in the hospital, and depression, pain catastrophizing, and anxiety on each POD.

aSMDs ≥ 0.2 were considered our lower bound of effect sizes [34,35]. R software (version 4.3.1, R Core Team, 2023) was used for data management and analysis. The *TOSTER* package was used to calculate SMDs, *DescTools* was used to calculate AUCs, and *halfmoon* was used to construct love plots.

## 3. Results

**Figure 1:**
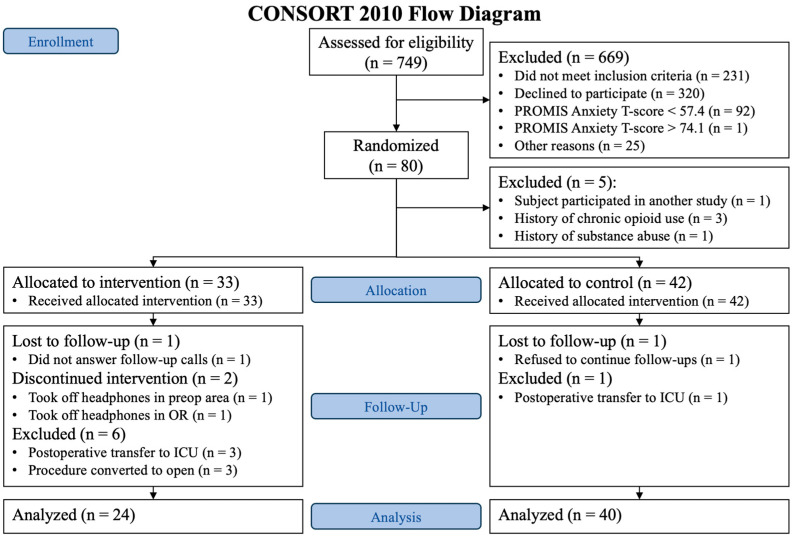
CONSORT flow diagram.

A total of 749 patients were screened from May 2022 to August 2023. We found 493 patients to be eligible; 173 gave informed consent, and 93 patients were considered screen failures because their PROMIS Anxiety T-scores were <57.4 or >74.1. Consequently, 80 patients were randomized. After randomization, five patients were found to be ineligible and removed from the final analysis: one patient participated in another study, one patient had a history of substance abuse, and three patients had a history of chronic opioid use. Out of the 75 remaining patients, 33 were randomized to the music-based intervention group and 42 to the control group. During the follow-up phase in the music-based intervention group, one patient was lost to follow-up, two patients withdrew from the study, and six were excluded due to postoperative transfer to the intensive care unit (ICU) and having their procedure converted from laparoscopic to open. In the control group, one patient was lost to follow-up, and one patient was transferred to the ICU after surgery (Figure 1).

Table 1 presents patients’ demographics, including age, sex, race, ethnicity, weight, height, body mass index, baseline PROMIS Anxiety, PROMIS Depression, and PCS scores, type of music being played, and the percentage of patients who filled their opioid prescription overall and in each group. These data indicated no observed statistical differences among groups at baseline.

Table 2 presents the types of surgery performed on patients included in the study. There were no observed significant differences between the two groups.

**Figure 2:**
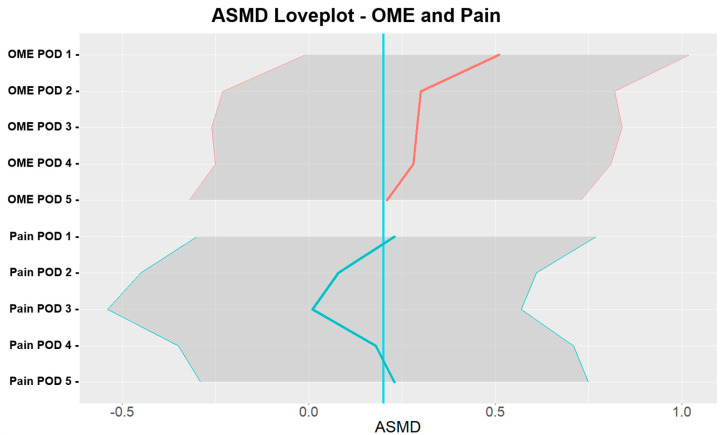
aSMD love plot for OME and pain in the intervention music therapy group vs. the control group. aSMD—absolute standardized mean difference; OME—oral morphine equivalent; POD—postoperative day. The blue line represents a reference for aSMD = 0.2.

Primary end point: As presented in Table 3 and Figure 2, patients in the intervention music therapy group consumed 56.7% less opioids than those in the control group (median 2.8 in intervention music therapy group vs. 6.4 control group, aSMD = 0.34; 95% confidence interval (CI) = (−0.17, 0.85)). While the estimated aSMD displayed an effect size ≥ 0.2, we noted that the CI contained zero. The greatest difference in opioid consumption was recorded on POD1 (0.51, (−0.01, 1.02)) and decreased after that. On POD5, opioid aSMD was 0.21 (−0.32, 0.73).

Secondary end point: The greatest difference in opioid consumption was recorded on POD1 (0.51, (−0.01, 1.02)) and decreased after that. On POD5, opioid aSMD was 0.21 (−0.32, 0.73). Pain scores using AUCs from POD1 and POD5 showed no difference between the groups (13.0 vs. 12.5; 0.12 (−0.4, 0.64)). Furthermore, our data demonstrated fluctuating effects of intervention music therapy on anxiety (POD2 0.38 (−0.16, 0.91)), depression (POD2 0.31 (−0.23, 0.84) and POD4 0.24 (−0.29, 0.77)), and pain catastrophizing (POD1 0.24 (−0.3, 0.77)). Table 4 presents the frequency of PONV, satisfaction scores, and length of hospital stay. aSMD sizes were small for satisfaction scores, frequency of PONV, and length of hospital stay (3.6 vs. 4.1 h; 0.11 (−0.4, 0.62)). Correlations between total post-op OME, depression, pain catastrophizing, and anxiety were negligible, with an absolute upper bound of 0.12.

**Figure 3:**
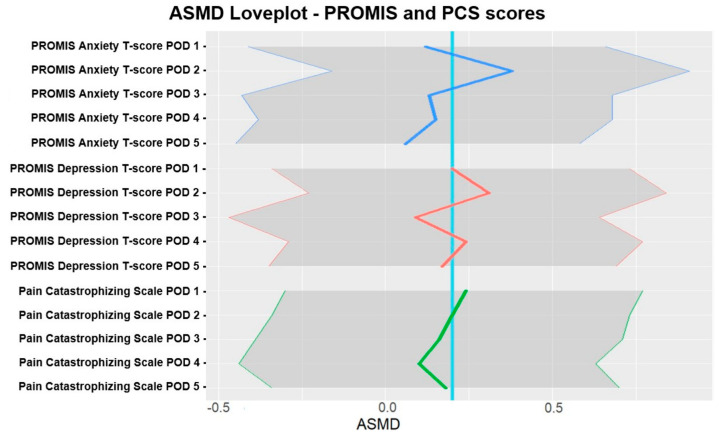
aSMD love plot for PROMIS and PCS scores in the intervention music therapy group vs. the control group. aSMD—absolute standardized mean difference; POD—postoperative day; PCS—Pain Catastrophizing Scale. The blue line represents a reference for aSMD = 0.2.

Figure 3 presents the difference in PROMIS Anxiety, PROMIS Depression, and PCS scores over five days based on aSMD and pain catastrophizing.

Table 4 presents the frequency of PONV, satisfaction scores, and length of hospital stay. aSMD sizes indicated no difference between the group for satisfaction scores, frequency of PONV, and length of hospital stay (3.6 vs. 4.1 h; 0.11 (−0.4, 0.62)). Finally, correlations between total post-op OME, depression, pain catastrophizing, and anxiety were negligible, with an absolute upper bound of 0.12.

## 4. Discussion

In contrast to other published studies [16,20,21,22,23,24,25,26,27,28], our study focused on the potential benefits of music-based intervention in patients with anxiety prior to surgery. Our data indicates that 46% of the patients included in this study had anxiety scores within the inclusion criteria. This finding is within the percentage of preoperative anxiety reported by Friedrich. In this context, females have been found to have higher levels of preoperative anxiety [36].

Our data suggest that music-based intervention may be an effective technique to reduce opioid requirements in patients undergoing minor surgery. Total opioid consumption for patients in the music-based intervention group was 56.7% lower than that for patients in the control group. The effect was the highest on POD1. Our data also suggest that by POD5, the difference between the groups was minimal (Figure 2), suggesting that in the context of our protocol (administrating music-based intervention prior to, during, and immediately after surgery), the effects of music-based intervention lasted four days. This short-lasting effect of music-based intervention may also be due to the fact that the role of music-based intervention was studied in patients undergoing minor surgery, where pain is expected to last no more than three to four days. [37]. Furthermore, while the aSMD indicated a noteworthy effect of 0.34, the associated 95% CIs (−0.17, 0.85) showed a large difference between the individual effects of music-based intervention on opioid consumption. Postoperative opioid consumption is multifactorial, including not only pain associated with the surgery, but also individual factors such as anxiety, depression, and catastrophizing.

The music protocol used in this study was developed by a licensed music therapist. Patients in this study were offered a choice of classical, jazz, piano, guitar, and lo-fi music. Our protocol is a significant deviation from those in other published studies on the use of music-based intervention in surgical patients. While it is uncertain whether our approach should serve as a reference for future studies, theoretically, involving a certified music therapist could enhance the benefits of music-based intervention in surgical patients.

Music-based intervention has been used in patients undergoing several types of surgery, including ambulatory and inpatient surgery, cancer and cardiac surgery, and total hip, knee, and shoulder arthroplasty. In most cases, music-based intervention has been administered after surgery to treat anxiety and depression and, in a limited number of cases, to reduce post-surgical pain. Juhl et al. (2019) [38] suggested that the mechanism of action of music-based intervention is multifactorial (cognitive function, emotion, and neurobiology). Our data provide additional evidence that music-based intervention is an interesting technique to not only decrease the anxiety and depression associated with surgery, but also decrease opioid requirements in the first five days following surgery. In our study, however, music-based intervention had minimal effects on pain.

Despite the reduction in opioid consumption, there was a lack of differences between the medians of the pain scores, most likely related to the intrinsic variability of the pain scores within each group that is reflected by the large aSMD 95% CI (−0.4, 0.64) and the relatively limited extent of the surgeries that were performed. Although we reported a reduction in both pain levels and postoperative opioid consumption associated with the use of aromatherapy and the NeuroCuple™ device [13,15], this absence of correlation between pain and total opioid consumption can be seen in other studies [39,40]. This absence of correlation might also be related to the temporal independence between the opioid intake and pain level recorded each day.

Secondary outcomes, including patient satisfaction, length of hospital stay, and PONV, were not substantially different between the groups. While music-based intervention may improve some aspects of recovery (such as reducing opioid consumption), it may not be sufficient to influence the overall length of hospital stay or PONV. Other factors, such as type of surgery, type of anesthesia, and the patient’s medical history, may be more determinant factors controlling these outcomes.

Our data suggest that music-based intervention reduced anxiety on POD2, depression on POD2 and POD4, and pain catastrophizing on POD1. While music-based intervention can provide temporary relief, it might be insufficient to consistently affect the emotional and cognitive aspects of these parameters during POD1-POD5, or PROMIS/PCS questionnaires are not designed to capture short-term mood changes. As for depression, the inconsistent effects could also be attributed to the low baseline scores in both treatment groups, showing no further decrease in already low scores.

Several limitations should be noted in our study. First, the sample size was relatively small, which may limit the generalizability of the findings. Second, although each patient was allowed to choose between five playlists with music from various genres, the researcher-selected music may not have covered the type of music preferred by a given patient. Lastly, the unequal distribution of patients between the placebo and the treatment groups may have affected the recorded overall responses between the groups.

Dang et al. [16] reported that the benefits of a combination of aromatherapy and music-based intervention were not greater than the benefits of either therapy alone. This suggests that before adding a combination of complementary techniques to a postoperative protocol to manage anxiety and depression in surgical patients, it is necessary to establish the benefit of that combination. This applies to the use of psychoeducation programs [41,42], acupuncture [43], auriculotherapy [44,45], hypnosis [46], virtual reality [47], aromatherapy [13], nanotechnology [15], and other potential complementary techniques, especially when the technique involves significant training, time, and cost, such as with acupuncture, auriculotherapy, and biofeedback.

In contrast to other published studies [16,20,21,22,23,24,25,26,27,28], our study focused on the potential benefits of music-based intervention in patients with anxiety prior to surgery. Our data indicate that 46% of patients in this study presented anxiety scores within the inclusion criteria. In this context, females have been found to have lower levels of preoperative anxiety [48].

Our study contributes to the growing body of evidence supporting the integration of music-based intervention into perioperative care protocols. Cost-effectiveness and ease of implementation make music-based intervention an attractive option for enhancing patient outcomes. However, variability in the observed effects highlights the need for further research to identify the patient populations that may benefit the most from this intervention.

## 5. Conclusions

Our study provides evidence to support the effectiveness of music-based intervention as a nonpharmacologic technique to reduce opioid consumption in the postoperative period. There was also a temporary effect on mood disorders. Future studies are necessary to confirm this concept. Further studies should include a larger sample size, assess whether or not music-based intervention should extend beyond the immediate perioperative period, and be applicable to surgeries beyond minor surgeries.

## Figures and Tables

**Table 1:** Baseline characteristics of study participants.

Variable		Total (*n* = 64)	Control (*n* = 40)	Treatment (*n* = 24)	*p* Value
Age, median (IQR)		54 (42.25–63)	56 (42.2–61.2)	49.5 (41.5–66.2)	0.961
Sex, No. (%)					0.778
	Female	45 (70.3%)	29 (72%)	16 (67%)	
	Male	19 (29.7%)	11 (28%)	8 (33%)	
Race, No. (%)					0.109
	Asian	3 (4.7%)	0 (0%)	3 (12%)	
	Black or African American	11 (17.2%)	7 (18%)	4 (17%)	
	White	48 (75%)	31 (78%)	17 (71%)	
	Other, not specified	2 (3.1%)	2 (5%)	0 (0%)	
Hispanic, No. (%)					0.137
	No	61 (95.3%)	39 (98%)	22 (92%)	
	Yes	2 (3.1%)	0 (0%)	2 (8%)	
	Not specified	1 (1.6%)	1 (2%)	0 (0%)	
Weight, median (95% CI) in kg		79.5 (67.5–98)	84 (67.5–99.5)	76 (67.7–97.7)	0.608
Height, median (95% CI) in cm		169 (160–175)	170 (160–175.1)	168 (159.4–173)	0.404
BMI, median in kg/m^2^ (95% CIU in kg/m^2^)		28.6 (24.8–33.5)	29.2 (24.6–33.8)	27.9 (52.2–32.9)	0.906
Music genre, No. (%)					
	Classical	3 (4.7%)		3 (12%)	
	Guitar	8 (12.5%)		8 (33%)	
	Jazz	9 (14.1%)		9 (38%)	
	Lo-Fi	2 (3.1%)		2 (8%)	
	Piano	2 (3.1%)		2 (8%)	
Opioid prescriptions filled, No. (%)					
	No	0 (0%)	0 (0%)	0 (0%)	
	Yes	33 (100%)	21 (100%)	12 (100%)	
Baseline PROMIS Anxiety T-score, median (95% CI)		62.5 (59.4–66.6)	62.5 (59.4–64.8)	63.5 (59.4–69)	0.452
Baseline PROMIS Depression T-score, median (95% CI		53.85 (49.8–57.9)	54.3 (49.8–57.9)	53.4 (50.9–57.5)	0.945
Baseline PCS score, median (95% CI)		14 (6.75–21.25)	16 (8–21.2)	12.5 (4–21.2)	0.532

CI (confident interval); BMI—body mass index; PCS—Pain Catastrophizing

**Table 2:** Types of surgical interventions among study participants.

Type of Surgery	Total (*n* = 64)	Control (*n* = 40)	Treatment (*n* = 24)	*p* Value
Mastectomy	9 (14.1%)	5 (12.5%)	4 (16.7%)	0.953
Breast reconstruction	17 (26.6%)	12 (30%)	5 (20.8%)	
Breast fat graft	2 (3.1%)	1 (2.5%)	1 (4.2%)	
Laparoscopic prostatectomy	12 (18.8%)	7 (17.5%)	5 (20.8%)	
Laparoscopic cholecystectomy	15 (23.4%)	10 (25%)	5 (20.8%)	
Laparoscopic appendectomy	3 (4.7%)	2 (5%)	1 (4.2%)	
Robotic inguinal hernia repair	3 (4.7%)	2 (5%)	1 (4.2%)	
Other laparoscopic procedures	3 (4.7%)	1 (2.5%)	2 (8.3%)	

**Table 3:** Pain score and opioid consumption AUC in the intervention music therapy group vs. those in the control group.

Variable	Control (*n* = 40)	Treatment (*n* = 24)	% Difference	aSMD (95% CI)
Pain score Median AUC for POD 1–5	12.5	13.0	4%	0.12 (−0.4, 0.64)
Opioid use in OME Median AUC for POD 1–5	6.4	2.8	−56.7%	0.34 * (−0.17, 0.85)

aSMD—absolute standardized mean difference; AUC—area under the curve; CI—confidence interval; OME—oral morphine equivalent; POD—postoperative day; * aSMD ≥ 0.2.

**Table 4:** Secondary outcome measures in the intervention music therapy group vs. the control group.

Variable	Control (*n* = 40)	Treatment (*n* = 24)	% Difference	aSMD (95% CI)
Satisfaction questionnaire score, median 95% CI))	10 (10–10)	10 (10–10)	0%	0.05 (−0.48, 0.58)
Time to hospital discharge, median in hours (95% CI)	4.1 (2.8–6.7)	3.6 (2.9–6)	−11.3%	0.11 (−0.4, 0.62)
Level of nausea, median (95% CI	0 (0–0)	0 (0–0)	-	0.22 (−0.29, 0.73)

aSMD—absolute standardized mean difference; CI—confidence interval.

## Data Availability

Data are available on http://www.ClinicalTrials.gov (NCT05263635).

## Appendix A

**Table A1:** Music by type (N/A = not available).

Genre	Song	Composer	Artist
CLASSICAL	Piano Concerto No. 5 in E-Flat Major, Op. 73	Ludwig van Beethoven	Vienna Philharmonic
	The Four Seasons Winter, op. 8/4, II Largo	Antonio Vivaldi	Stuttgart Chamber Orchestra
	Piano Concerto No 21 in C Major, K. 467	Wolfgang Amadeus Mozart	Numberg Symphony Orchestra
	Romance for Violin and Orchestra in F Major, Op 50	Ludwig van Beethoven	Badische Staatskapelle
	Suite for Orchestra No. 3 in D Major	Johann Sebastian Bach	Mainz Chamber Orchestra
	L’estro Armonico No. 8 in A minor, Op. 3	Antonio Vivaldi	Stuttgart Chamber Orchestra
	Andagio for Strings (from Strings Quartet, Op 11)	Samuel Barber	New Zealand Symphony Orchestra
	Ellen’s Song III (“Ave Maria”), D. 839 (Op 52/6)	Franz Schubert	Aaron Rosand & Eileen Flissier
	Symphony No..6 in B Minor, Op. 74	Pyotr Ilyich Tchaikovsky	Slovak Philharmonic Orchestra
	Symphony No. 6 in F Major, Op 68	Ludwig van Beethoven	London Symphony Orchestra
	Piano Concerto No. 2 in C Minor, Op 18	Sergei Rachmaninoff	Moscow RTV
	Piano Concerto No. 1 in E minor, Op 11	Frederic Chopin	Hamburg Symphony Orchestra
	Violin Concerto in D Major, Op 35	Pyotr Ilyich Tchaikovsky	Orchestra of Radio Luxemburg
	Violin Concerto in D Minor for 2 Violins and Orchestra	Johann Sebastian Bach	Mainz Chamber Orchestra
	Symphony No 5 in C-Sharp Minor: IV. Adagietto	Gustav Mahler	London Symphony Orchestra
	Songs Without Words, Op 62: Spring Song	Felix Mendelssohn	Hans Kalafusz & Klaus von Wildemann
	Concerto No. 20 in D Minor for Piano and Orchestra	Wolfgang Amadeus Mozart	Berlin Symphony Orchestra
	Concerto No. 3 in G Major for Violin and Orchestra	Wolfgang Amadeus Mozart	Dalibor Brazda
	Carnival of the Animals: XIII The Swan	Camille Saint-Saens	Stockholm Chamber Duo
	Thais Meditation (Andante Religioso)	Jules Massenet	Royal Philharmonic Orchestra
	String Quartet No 2 in D Major: Notturno	Alexander Borodin	Lucerne String Quartet
	Holberg Suite in G Major, Op 40: Sarabande	Edvard Grieg	Slovak Philharmonic Orchestra
	Suite Bergamasque, L 75: Clair de lune	Claude Debussy	Mostar Symphony Orchestra
PIANO	Suite Bergamasque: III. Clair de Lune	Claude Debussy	Peter Frankl
	Reverie, for Piano, L. 68	Claude Debussy	Peter Frankl
	Scenes from childhood, Op 15: VII Traumerei	Robert Schumann	Peter Schmalfuss
	12 Etudes, Op. 10: No. 3 in E major	Frederic Chopin	Abbey Simon
	Piano Concerto No 2 in C Minor, Op 18	Sergei Rachmaninoff	Moscow RTV
	Piano Concerto No 1 in E Minor, Op. 11	Frederic Chopin	Hamburg Symphony Orchestra
	Piano Concerto No. 5 in E-Flat Major, Op. 73	Ludwig van Beethoven	Vienna Philharmonic
	3 Gymnopedies: Gymnopedie No 1	Erik Satie	Frank Glazer
	Melodies for Piano, Op 3: No 1 Melody In F	Anton Rubinstein	Michael Ponti
	Waltzes, Op 39: No. 15 in A major	Johannes Brahms	Alfred Bredela dn Walter Klien
	Sonata for piano No. 12 in F Major, K 332	Wolfgang Amadeus Mozart	Carmen Piazzini
	Nocturnes, Op. 27, No. 2 in D-flat major	Frederic Chopin	Abby Simon
	Concerto No. 20 in D Minor for Piano and Orchestra	Wolfgang Amadeus Mozart	Berlin Symphony
	12 Preludes, Book 1, L 117	Claude Debussy	Peter Schmalfuss
	Trois Gymnopedies: Gynopedie No. 1	Erik Satie	Frank Glazer
	Aria and 30 Variations	Johann Sebastain Bach	Henrik Mawe
	Amazing Grace	John Newton William Walker	Relaxing Piano
	Sekai no Shazo kara	Nakamura Hachidai	Relaxing Piano
	Amaratine	Enya Roma Ryan	Relaxing Piano
	Music Room After School	Gonititi Masayo	Relaxing Piano
	My Favorite Things/Sound of Music	Rodgers Hammerstein	Relaxing Piano
	Give Me a Wing	Kunihiko Murai	Relaxing Piano
	Reprise/Spirited Away	Joe Hisaishi	Relaxing Piano
	ALWAYS—Sunset on Third Street	Naoki Sato	Relaxing Piano
	You Raise Me Up	Brendan Graham Rolf Lovland	Relaxing Piano
	Comme au Premier Jour	Ande Gagnon Doug Gamely	Relaxing Piano
	Etupirka	Taro Hakase	Relaxing Piano
	TAKUMI	Masa Takumi	Relaxing Piano
	KAZABUE	Michiru Oshima	Relaxing Piano
	JIN—Main Title	Yuu Takami	Relaxing Piano
	Let It Go/Frozen	Kristen Anderson Lopez Robert Lopez	Relaxing Piano
	Nuovo Cinema Paradiso Tema D’Amore	Andrea Morricone Ennio Morricone	Relaxing Piano
	Merry Christmas Mr. Lawrence	Ryuichi Sakomoto	Relaxing Piano
	Kaze no Uta	Toshihiko Sahashi	Relaxing Piano
	Flowers Will Bloom	Yoko Kanno	Relaxing Piano
	Time to Say Good-Bye	Francesco Sartori. Lyricist: Frank Peterson. Lucio Quarantotto	Relaxing Piano
	Lost	Jacky Terrasson	Wallace Roney
GUITAR	Gymnopedie No. 1	Erik Satie	Robert Lunn
	Clair de Lune	Claude Debussy	Tariq Harb
	Cannon in D	Johann Pachelbel	Ines Thome
	Gymnopedie No. 3	Erik Satie	Micharl Christian Durrant
	Jeux Interdits (Forbidden Games)	Narciso Yepes	Paco Hernandes
	Guitar Concerto de Aranjuez Rodrigo	Joaguin Rodrigo	Julian Bream
	Air Jean Baptiste Lully	Jean Lully	Richard Mollenbeek
	Pavane Faure	N/A	Pablo Segovia Gardel
	Cavatina (Deer Hunter Theme)	Stanky Myers	Mason Wilson
	Summertime	Gershwin Heywaard Kuhns	Julio Deranjo
	Dance of the Miller	Manuelde Falla	Juan Iniesta
	Gran Vals	Francisco Tarrega	Hegovia Juanrez
	Una Furtiva Lagrima	Gaetano Donizetti	Pablo Segovia Gardel
	Gymnpedie	Erik Satie	Francisco Tores
	Malaguena	Ernesto Lecuona	Gypsy Queens
	El Mariachi (Once Upon a time in Mexico)	Robert Rodriguez	Leo Sanchez
	Moonlight Sonata	Beethoven	Rodrigo Escoba
	Valse Criollo	Antonio Lauro	Neo Yepes
	Bolero	Ravel	Gipsy Rayes
	Granada (Suite Espanola)	Isaac Albeniz	Ricardo Juarez
	Prelude, No. 5	Heitor Villa Lobos	Rodrigo Escoba
	Albinoni arr Giazotto: Adagio in G Minor	Tsomaso Albinoni & Remo Giazotto	Dominic Miller, Budapest Film Orchestra
	Myers: Cavatina (The Deer Hunter)	Stanley Myers	Goran Sollscher
	Guitar Concerto in D—Largo	Antonio Vivaldi	Eduardo Fernandez
	Cello Suite No. 1—Prelude	Johann Sebastian Bach	John Williams
	Apres un reve	Gabriel Faure	Steve Erquiaga
	Pavane pour une infante defunte	Maurice Ravel	Steve Erquiaga
	Cello Suite No. 3—Courante	Johann Sebastian Bach	Pepe Romero
	Castillos de Espana—Torija	Federico Moreno torroba	Andres Segovia
	Prelude No. 1	Heitor Villa-Lobos	Julian Bream
	Andaluza	Enrique Granados	Pepe Romero & Celine Romero
	Suite espanola—Granada	Isaac Albeniz	Pepe Romero
	Bachianas brasileiras No. 5, W. 389	Heitor Villa-Lobos	Aleandre Lagoya
	Cello Concerto No.6 Andante	Luigi Boccherini	Andres Segovia
	2 Temas Populares Cubanos	Leo Brouwer	Eduardo Fernandez
	Cantana No 156—Arioso	Johann Sebastian Bach	Steve Erquiaga
	Romane d’amour	Anonymous	goran Sollscher
	Mass in B Minor—Qui Tollis	Johann Sebastian Bach	Dominic Miller, Budapest Film Orchestra
	Twin Peaks Theme	Angelo Badalamenti	Henrik Janson
	Mad World	Roland Orzabal	Sergei Baronin
	Be Over Ay	Shazam Footer	John Hanks
	Cornfield Chase	Hans Zimmer Alex Gibson	Moldoni
	Chi Mai	Ennio Morricon	Henrik Janson
	Bibo No Aozora	Ryichi Sakomoto	Henrik Janson
	Memories (Top Gun)	Harold Falter-Meyer	Christopher Varela
	Deep Forest	Omar Franco Varela	Christopher Varela
	Adagio	Henrik Janson	Richard Mollenbeck
	Gymnopedie	Erik Satie	Richard Mollenbeck
	Hills and Horizons	Evans, Rolls, Leslie and Barlow	John Hanks
	Lost and Found	William Puchot	John Hanks
	Pavane, Op. 50	Fauve	Marcel Dopuis
	New Moon (The Meadow)	Alexandre Desplat	Miranda Boumedin
	Ave Nocturne	John Field	John A. Nilson
	Theme from Schindler’s List	John Williams	Moldoni
	Pathetique Sonata	Beethoven	Marcel Dopuis
	Air on a G String	J. S. Bach	Richard Mollenbeck
	Carrousel	Rogers/Hammerstein	N/A John A. Nilson
	In Trutina	Carl Orff	Marcel Dopuis
	May it Be	Enya/Ryan	Sergei Baronin
	Sweden	Getz/Hallberg	Sergei Baronin
	River Flows in You	Yiruma	Miranda Boumedin
	In My Spanish Hacienda	Rodrigo	Sergio Miguel
	The Ludlows	J. Horner	Christopher Varela
	Vivaldino	A. Carrilho	John A. Nilson
	Braids	Paterson	Enrico Carmona
	Transformation	E Menken	Enrico Carmona
JAZZ	Movement I, Pt. I	Bob Belden and Suzanne Severini	Classical Jazz Quartet
	Movement I, Pt. III	Bob Belden and Suzanne Severini	Classical Jazz Quartet
	Movement I, Pt. IV	Bob Belden and Suzanne Severini	Classical Jazz Quartet
	Movement II, Pt. II	Bob Belden and Suzanne Severini	Classical Jazz Quartet
	Round Midnight	Thelonious Monk and Cootie Williams	Hank Jones
	Yesterdays	Jerome Kern Otto Harbach	Larry Coryell
	Into the Shadows	John Fedchock	James Moody
	Second Time Around	Sammy Cahn-Jimmy Van Heusen	Donald Brown
	Yvette	Gigi Gryce	Darrell Grant
	Sunny	Bobby Hebb	Les McCann
	Sweet & Lovely	Gus Arnheim, Charles N. Daniels and Harry Tobias	Sonny Stitt
	A Flower	Kenny Barron	Kenny Barron
	My Man’s Gone Now	George Gershwin DuBose Heyward	Mulgrew Miller
	When You Wish Upon a Star	Leigh Harline Ned Washington	Eliane Elias
	Nature Boy	Eden Ahbez	The Drummonds
	This Guy’s In Love with You	Burt Bacharach Hal David	Cedar Walton
	Lover Man	Jimmy Davis, Roger Ramirez, James Sherman.	Sonny Stitt
	You Better Go Now	Irvin Graham Bix Reichwer	Red Garland
	Willow Weep For Me	Ann Ronel	Cannonball Adderley
	Love Walked In	George Gershwin	Errol Garner
	Summertime	George Gershwin	Duke Jordan
	Moonlight in Vermont	John Blackburn Karl Suexxdorf	Marian McPartland
	When Darkeness Falls	George Shearing	George Shearing
	Solace	S. Joplin	Billy Taylor
	Softly as in a Morning Sunrise	Romberg/Hammerstein	The modern Jazz Quartet
	Blue in Green	Davis/Evans	Miles Davis
	Flamenco Sketches	Davis/Evans	Miles Davis
	Fran-Dance	Davis	Miles Davis
	Stella By Starlight	Young/Washington	Miles Davis
	The Waking Hours	A. Holzwarth	Jeff Bailey
	Sweet Sadness	Clary/Bottini	Jeff Bailey
	The Only One for Me	McKnight	Jeff Bailey
	Precious Few	Kent	Jeff Bailey
	Beauty Marks	S. Grey	Jeff Bailey
	Make Believe Waltz	Morse/Drislane	Jeff Bailey
	Memories	E. Blake	Jeff Bailey
	In This Place	T. Thompson	Jeff Bailey
	Open Road	Williamson	Jeff Bailey
	After Hours	A. Parrish	Jeff Bailey
	Lullaby for Lovers	B. Kaempfert	Jeff Bailey
LO-FI	Dreamy Vibe	Loonight, clava & kazuna	Loonight, clava & kazuna
	Good Night	Loonight, clava & kazuna	Loonight, clava & kazuna
	Peaceful	Loonight, clava & kazuna	Loonight, clava & kazuna
	Sleepy Tune	Loonight, clava & kazuna	Loonight, clava & kazuna
	Sunset Love	Loonight, clava & kazuna	Loonight, clava & kazuna
	Blue Bottle	Blue Tumbler	Lofi Blue Tumb
	Starbucks	LofiGuy	Lofi LofiGuy
	Taylor Coffee	LofiGuy	Lofi LofiGuy
	MONACLE	Bruns	Lofi Bruns
	% Arabica	Lost Tribe	Lofi Lost Tribe
	Intelligentsia	LofiGuy	Lofi LofiGuy
	TERAROSA	LofiGuy	Lofi LofiGuy
	DOUTOR	LofiGuy	Lofi LofiGuy
	Daydreams	Pink Marble	Lofi Pink Marble
	Walk in the Blue Mountains	Kitsune	Lofi Kitsune
	Endless	LofiBeats	Lofi LofiBeats
	Atmosphere	L. Walther	Lofi L. Walther
	Nostalgia	Glimlip Sleepermane	Lofi Glimlip Sleepermane
	Revenant	Medieval Lofi	Lofi Medieval Lofi
	Autumn Breeze	Annata A.I./Zen Vibes	Lofi Annata A.I./Zen Vibes
	Inception	Hans Zimmer	Lofi Ender Guney
	Chillin in my House	BLK (Kor)	Lofi Antique Sound Lab
	Coffee and Cake	ILLIN	Lofi Antique Sound Lab
	Song for Lazy bone	ILLIN	Lofi Antique Sound Lab
	Smoking After Lunch	ILLIN	Lofi Antique Sound Lab
	Sunset	Lukrembo	Lofi Lukrembo
	Ocean Sunset	chiro17, Riinholm & Magnoshi Kato	chiro17, Riinholm & Magnoshi Kato
	Campfire	chiro17, Riinholm & Magnoshi Kato	chiro17, Riinholm & Magnoshi Kato
	Friday	chiro17, Riinholm & Magnoshi Kato	chiro17, Riinholm & Magnoshi Kato
	Sunrays	chiro17, Riinholm & Magnoshi Kato	chiro17, Riinholm & Magnoshi Kato
	Don’t Worry	chiro17, Riinholm & Magnoshi Kato	chiro17, Riinholm & Magnoshi Kato
	Feeling Blue	Menda, loonight & clava	Menda, loonight & clava
	Ocean Waves	Menda, loonight & clava	Menda, loonight & clava
	Beach Day	Menda, loonight & clava	Menda, loonight & clava
	Sunset	Menda, loonight & clava	Menda, loonight & clava
	Butterly	Menda, loonight & clava	Menda, loonight & clava
	Dim the Lights	Jamania, Menda & Knoodle	Jamania, Menda & Knoodle
	Break of Dawn	Jamania, Menda & Knoodle	Jamania, Menda & Knoodle
	Sunset Love	Jamania, Menda & Knoodle	Jamania, Menda & Knoodle
	So Good	Jamania, Menda & Knoodle	Jamania, Menda & Knoodle
	Vacation	Jamania, Menda & Knoodle	Jamania, Menda & Knoodle
	Hallucination	Lars Narvike	Lars Narvike
	Journey to Jordan	Lars Narvike	Lars Narvike
	Mikawa	Lars Narvike	Lars Narvike
	Woods of Love	Lars Narvike	Lars Narvike
	Sunshine in my heart	finton, Chillski & Narvike	finton, Chillski & Lars Narvike
	Golden Hour	finton, Chillski & Narvike	finton, Chillski & Lars Narvike
	Peanut Butter Jelly	finton, Chillski & Narvike	finton, Chillski & Lars Narvike
	Moonlight	finton, Chillski & Narvike	finton, Chillski & Lars Narvike
	The Journey	finton, Chillski & Narvike	finton, Chillski & Lars Narvike
	Quiet Thoughts	clava	clava
	Anymal	clava	clava
	Head to Head	clava	clava
	Nights in Rome	clava	clava
	Part of This	Bristic, snaate & Riinholm	Bristic, snaate & Riinholm
	Bar Night	Bristic, snaate & Riinholm	Bristic, snaate & Riinholm
	Dinner for Two	Bristic, snaate & Riinholm	Bristic, snaate & Riinholm
	Our History	Bristic, snaate & Riinholm	Bristic, snaate & Riinholm
	Love Story	Bristic, snaate & Riinholm	Bristic, snaate & Riinholm
	Sao Paolo Sunset	snaate	Snaate
	I Miss Home	snaate	Snaate
	Good Reputation	snaate	Snaate
	Passion	snaate	Snaate
	Wonderful Life	kazuna	Kazuna
	Waves	kazuna	Kazuna
	Morning Flow	kazuna	Kazuna
	Spring	kazuna	Kazuna
	Sunday Stroll	kazuna	Kazuna
	Call Me Please	Lars Narvike	Lars Narvike
	Got the Feels	Lars Narvike	Lars Narvike
	Salir De Marcha	Lars Narvike	Lars Narvike
	Un Flechazo	Lars Narvike	Lars Narvike
	Happy Times	Jamania	Jamania
	Paris	Jamania	Jamania
	Dinner for One	Jamania	Jamania
	In the Moment	Jamania	Jamania
	Daydreaming	Jamania	Jamania
	Blossom	Chillski	Chillski
	New York Nights	Chillski	Chillski
	Diverted	Chillski	Chillski
	Dreamy Clouds	Chillski	Chillski
	Happy Day	Chilllski	Chillski
	Blue Sky	Jamania	Jamania
	Friendship	Jamania	Jamania
	Clouds	Jamania	Jamania
	Lucky Day	Jamania	Jamania
	Late Night Drive	Jamania	Jamania
